# Epidemiology of menthol cigarette use in the United States

**DOI:** 10.1186/1617-9625-9-S1-S1

**Published:** 2011-05-23

**Authors:** Ralph S Caraballo, Katherine Asman

**Affiliations:** 1 Mail Stop K-50, Office on Smoking and Health, National Center for Chronic Disease Prevention and Health Promotion, Centers for Disease Control and Prevention, 4770 Buford Highway, N.E., Atlanta, GA, USA; 2 Research Triangle Institute (RTI) International, Koger Center, Oxford Building, Suite 119, 2951 Flowers Road South, Atlanta, GA, USA

## Abstract

**Background:**

Approximately one-fourth of all cigarettes sold in the United States have the descriptor “menthol” on the cigarette pack. It is important to determine what socio-demographic factors are associated with smoking menthol cigarettes if indeed these types of cigarettes are related to smoking initiation, higher exposure to smoke constituents, nicotine dependence, or reduced smoking cessation.

**Methods:**

The National Cancer Institute (NCI) conducted a review of the scientific literature on this topic which we completed by adding more recently published articles via PubMed. We also conducted further data analyses using the National Survey on Drug Use and Health, the National Youth Tobacco Survey, the Monitoring the Future Survey, and the National Health and Nutrition Examination Survey to provide up-to-date information on this topic.

**Results:**

Menthol cigarettes are disproportionately smoked by adolescents, blacks/African Americans, adult females, those living in the Northeast of the United States and those with family incomes lower than $50,000. Based on self-reports of menthol cigarette use, menthol cigarette use among smokers have increased from 2004 to 2008. However, no increase was observed during these years for predominantly menthol brands like Newport™, Kool,™ and Salem™, however, this lack of significant trend may be due, at least in part, due to smaller numbers of smokers of specific brands or sub-brands, which provide estimates which are less precise.

**Conclusion:**

Menthol cigarettes are disproportionately smoked by groups of U.S. cigarette smokers. It is likely that other disparities in menthol cigarette use exist that we have not covered or have not been studied yet.

## Background

There are over a thousand cigarette brands and sub-brands that are sold in the United States [1]. Cigarette brands are advertised or described by some specific characteristics such as been filter or non-filter, by the length of the cigarette, by its Federal Trade Commission machine-measured levels of tar, nicotine yield, and carbon monoxide, and some brands of cigarettes have the descriptor “menthol” in the pack [2].

It has been proposed that the anesthetic and cooling sensation properties of menthol allow smokers of menthol cigarettes (by menthol cigarettes we mean a cigarette from a pack with the descriptor “menthol” in it) to inhale more smoke from each cigarette than smokers of nonmenthol cigarettes (no “menthol” descriptor in the cigarette pack) [3,4]. It has also been proposed that smokers of menthol are more nicotine dependent, and as a consequence, are less likely to quit [5]. It has been hypothesized that the resulting higher smoke exposure over time may result in higher smoking-related diseases among smokers of menthol cigarettes [5,6]. In the U.S., menthol brands tend to have higher yields of tar, nicotine, and CO, however, menthol is present at reduced levels in many nonmenthol brands [7]. Finally, it has been proposed that menthol levels in cigarettes may play an important role in smoking initiation especially among adolescents, most of whom are underage [8].

In this article, we used a recent literature review by The National Cancer Institute (NCI) to determine what socio-demographic factors are associated with smoking (e.g., initiation, cessation, exposure to smoke constituents, nicotine dependence) menthol cigarettes and we completed it by adding more recently published articles we identified via PubMed. We also conducted data analyses with several national data sets to provide up-to-date information on this topic.

## Methods

Because type of cigarettes smoked, including brand preference, varies by age group [9,10], data on socio-demographic factors associated with smoking menthol cigarettes will be presented separately for youth and adults. A review of the scientific literature on socio-demographic factors related to smoking menthol cigarettes was provided by the Department of Health and Human Services’ National Cancer Institute (NCI) [11]. For youth, there were nine articles listed in the bibliography, of which one was conducted in Japan [12]. Of the eight studies conducted in the U.S., five [13-17] collected data using national samples, two were conducted in specific communities [18,19], and one study [20] was qualitative (focus groups) in study design. Four of these 8 U.S. articles presented data from cross-sectional studies [13,14,17,18], two from a longitudinal study [15,16], and two from a convenience sample [19,20]. Because more recent information has been published or available since the NCI bibliography was provided, additional studies or reports [8,10,21] are included in the youth section in this article. When appropriate, we report results from these studies in this review article.

For adults, there were 15 articles listed and one letter to the editor on the bibliography provided by NCI. Eleven of these 15 articles presented data from cross-sectional studies, four presented data from case-control studies or data were collected using convenience samples. The most recent study listed [22] reported data collected in 2002 from a specific U.S. population group (heroin users) in a specific location. Because of lack of recent data in the NCI bibliography, other studies and reports [8,10,23,24] were included in the adult section to provide more recent information on this topic. Also when appropriate, we report results from these studies in this review article.

To provide up-to-date information on this topic, data analyses were performed using specific data sets such as the *National Survey on Drug Use & Health*[25], hence referred as NSDUH; the *National Youth Tobacco Survey*[26], hence referred as NYTS; the *Monitoring the Future Survey*[27], hence referred as MTFS; and the *National Health and Nutrition Examination Survey*[*28*], hence referred as NHANES. It is important to note that the consistent collection of national data on menthol cigarette use started fairly recently, in the late 1990’s (MTFS) or in the early 2000’s (NSDUH, NYTS, and NHANES). Two-sided t tests were used to assess differences between population group percentages. For all tests, p<0.05 was considered statistically significant.

### NSDUH

The National Survey on Drug Use and Health (NSDUH) is a nationwide household survey that collects data on drug use and drug abuse, including tobacco use, from a representative sample of the U.S. civilian, noninstitutionalized population aged 12 years or older. Specifically, the NSDUH collects data on overall tobacco use, cigarette smoking, and other behavioral information related to cigarette smoking and brand preference. NSDUH data are collected through a computerized questionnaire administered in the privacy of participants’ homes by a professional field interviewer who visits each selected household. Most responses are answered in private by the participant, although the interviewer reads and enters the responses to some questions in the presence of the participant. Questions about tobacco use were administered through audio, computer-assisted, self-interview methods to maximize privacy and improve reporting of sensitive behaviors. For our analysis using these data, we used information for adolescents aged 12-17 years old who smoked in the past month (N=9,595) and adult smokers (aged 18 years or older) who smoked in the past month (N=62,010) from the 5 surveys conducted in 2004, 2005, 2006, 2007, and 2008 in order to determine prevalence of menthol cigarette use in the overall population of smokers as well as for specific subgroups of smokers and to assess trends in smoking menthol cigarette use among smokers.

### NYTS

The NYTS is a nationally representative sample of students enrolled in grades 6 through 12. The sampling universe consists of public and private school students in the 50 states and the District of Columbia. The sampling frame stratified the 50 states and the District of Columbia by region and urbanicity. Primary sampling units (PSUs), are selected with probability proportional to the student enrollment in the PSU but giving disproportionate weight to Black, Asian, and Hispanic students.

Schools are grouped by size as either large or small, depending on whether they have at least 125 students combined in eligible grades. All students present in a selected classroom on the day of the interview are selected for the study. Schools or students who refused to participate in the study are not replaced in the sample.

Our analysis included 1,978 middle school students and 6,163 high school students from year 2004, 2006, and 2009 combined who had valid information on the school year, past 30 day smoking, brand use, and menthol questions. Those who were excluded included: those who did not specify a grade in school or didn’t answer the question (n=397); those who were not a current smoker or didn’t answer the question (n=67,809); those who said they didn’t smoke cigarettes during the past 30 days, that they did not have a usual brand when asked about brand use, or did not answer the question (n=66,719); and those who said they do not smoke cigarettes when asked about menthol cigarettes or did not answer the question (n=59,965).

We analyzed data from 2,580 adolescent smokers selected throughout 35 states (not all states are represented because the survey design did not control for this) and from 267 large and small private and public schools.

### MTFS

The Monitoring the Future Survey (MTFS) main data collection involves a series of large, annual surveys of nationally representative samples of public and private secondary school students in grades 8^th^, 10^th^, and 12^th^ throughout the coterminous United States. Staff members administer the questionnaires to students, usually in their classrooms during a regular class period. Participation is voluntary. Parents are notified well in advance of the survey administration and are provided the opportunity to decline their child’s participation. Questionnaires are self-completed. For the combined years of 1998 to 2008, there were 20,863 8^th^ grade current smokers, 30,722 10^th^ grade current smokers, and 40,914 12^th^ grade current smokers in our data analysis. We assessed trends in smoking menthol cigarettes among adolescent smokers.

### NHANES

The National Health and Nutrition Examination Survey (NHANES) consists of a number of questionnaires administered in the household followed by standardized physical examinations and additional tobacco use questions administered in specially equipped mobile examination centers (MECs). The NHANES target population is the civilian, noninstitutionalized U.S. population. This nationally representative sample permits calculation of national estimates. NHANES over-samples low-income persons, adolescents 12–19 years, persons 60+ years of age, blacks, and Mexican Americans. We used NHANES data collected between January 2001 and December 2006. The overall response rate to NHANES for 2003**–**2008 was 78%. The analytic sample for this study included smokers aged 20 years and older who had smoked, who were recoded by NHANES as non-Hispanic white, non-Hispanic black/African American, or Mexican American. Of the 14,272 white, black, or Mexican American adults aged 20 years and older who completed the NHANES home interview. The final analytic sample included 2,319 individuals, of which 1,581 showed the 8 or 12 digit Universal Product Code (UPC) information on the side of the cigarette pack.

## Results

### Youth

**Figure 1:**
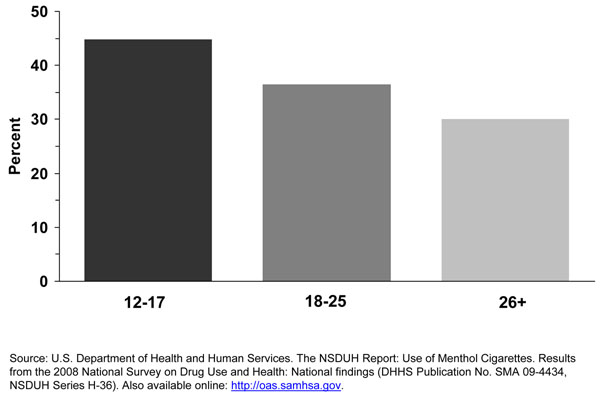


In the combined years 2004 to 2008, almost half of adolescent smokers aged 12 – 17 years reported smoking menthol cigarettes [10], an estimated 1 million adolescents (Table 1). Self-reports of types of cigarettes smoked are subject to bias. This important topic will be discussed in more detail later. Younger smokers are more likely to smoke menthol cigarettes than older smokers [10,16]. Figure 1 shows that a higher proportion of cigarette smokers smoked menthol cigarettes among adolescents (44.8%) than among young adults aged 18-25 years (36.5%) or older adults (30.1%). Consistent with this result specifically for adolescent current smokers, data for years 2004, 2006, and 2009 in the NYTS survey shows that 49.4% of middle school current smokers and 44.9% of high school current smokers reported smoking menthol cigarettes (Table 2). Finally, Aplleyard and colleagues found in a school-based survey using 2000 NYTS data that 42.0% of high school smokers who smoked in the past 30 days reported to have smoked a menthol brand [11].

**Table 1:** Prevalence for menthol by gender and age, NSDUH 2004-2008, by Year

YR	Age	GENDER	Menthol	Row Percent	Lower 95% Limit ROWPER	Upper 95% Limit ROWPER	Sample Size	Weighted Size
2004	12-17	Male	Menthol Cigarettes	40.35	36.44	44.39	410	560703
			Non-menthol cigs	59.65	55.61	63.56	609	828727
		Female	Menthol Cigarettes	46.26	42.07	50.5	530	665927
			Non-menthol cigs	53.74	49.5	57.93	605	773719
2005	12-17	Male	Menthol Cigarettes	37.09	32.82	41.59	382	485098
			Non-menthol cigs	62.91	58.41	67.18	633	822642
		Female	Menthol Cigarettes	46.14	41.55	50.8	490	586310
			Non-menthol cigs	53.86	49.2	58.45	593	684353
2006	12-17	Male	Menthol Cigarettes	40.41	35.38	45.65	379	505145
			Non-menthol cigs	59.59	54.35	64.62	569	744819
		Female	Menthol Cigarettes	47.05	43.17	50.97	420	603299
			Non-menthol cigs	52.95	49.03	56.83	506	678935
2007	12-17	Male	Menthol Cigarettes	46.93	42.96	50.93	460	586303
			Non-menthol cigs	53.07	49.07	57.04	511	663058
		Female	Menthol Cigarettes	50.24	46.52	53.97	416	560236
			Non-menthol cigs	49.76	46.03	53.48	415	554793
2008	12-17	Male	Menthol Cigarettes	48.35	43.88	52.84	383	510494
			Non-menthol cigs	51.65	47.16	56.12	467	545410
		Female	Menthol Cigarettes	48.2	43.86	52.57	398	496226
			Non-menthol cigs	51.8	47.43	56.14	419	533267

**Table 2:** Gender differences for menthol use, NYTS 2004, 2006, 2009

YR	School	Gender	Menthol	Row Percent	Lower 95% Limit ROWPER	Upper 95% Limit ROWPER	Sample Size	Weighted Size	p-Value
2004	Middle School	Female	All	100	.	.	554	470,743	0.5465
			Menthol	49.3	44.19	54.43	282	232,081	
			Non-menthol	50.7	45.57	55.81	272	238,661	
		Male	All	100	.	.	517	409,789	
			Menthol	47.94	44	51.9	262	196,437	
			Non-menthol	52.06	48.1	56	255	213,352	
	High School	Female	All	100	.	.	1214	1,459,070	0.001
			Menthol	49.38	42.06	56.73	614	720,532	
			Non-menthol	50.62	43.27	57.94	600	738,538	
		Male	All	100	.	.	1290	1,360,050	
			Menthol	39.14	34.02	44.51	552	532,345	
			Non-menthol	60.86	55.49	65.98	738	827,705	
2006	Middle School	Female	All	100	.	.	350	363,257	0.0732
			Menthol	42.54	36.73	48.57	157	154,529	
			Non-menthol	57.46	51.43	63.27	193	208,727	
		Male	All	100	.	.	367	329,602	
			Menthol	50.11	45.31	54.91	187	165,174	
			Non-menthol	49.89	45.09	54.69	180	164,427	
	High School	Female	All	100	.	.	1102	1,275,510	0.0066
			Menthol	44.82	37.05	52.86	541	571,688	
			Non-menthol	55.18	47.14	62.95	561	703,822	
		Male	All	100	.	.	1304	1,367,304	
			Menthol	37.8	32.74	43.14	544	516,841	
			Non-menthol	62.2	56.86	67.26	760	850,463	
2009	Middle School	Female	All	100	.	.	232	245,933	0.5966
			Menthol	52.97	47.07	58.79	121	130,275	
			Non-menthol	47.03	41.21	52.93	111	115,658	
		Male	All	100	.	.	277	327,945	
			Menthol	55.37	45.71	64.63	142	181,569	
			Non-menthol	44.63	35.37	54.29	135	146,376	
	High School	Female	All	100	.	.	935	1,070,338	0.0001
			Menthol	54.28	47.71	60.69	508	580,941	
			Non-menthol	45.72	39.31	52.29	427	489,396	
		Male	All	100	.	.	1188	1,433,809	
			Menthol	45.78	41.53	50.09	574	656,388	
			Non-menthol	54.22	49.91	58.47	614	777,421	

**Figure 2:**
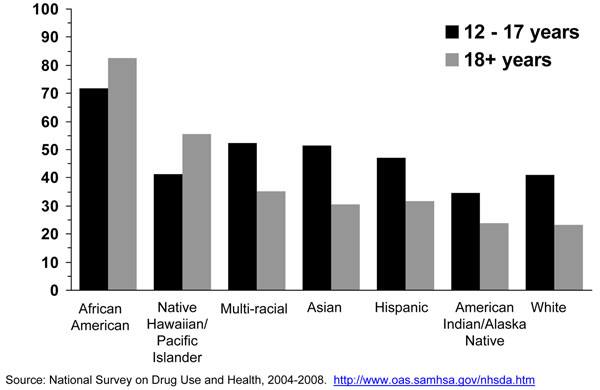


The proportion of menthol smokers among all cigarette smokers is higher among adolescents than among adults in most, but not all, racial or ethnic groups (Figure 2). Among white, multi-racial, Asian, and Hispanic youth, the proportions of adolescent cigarette smokers reporting smoking menthol cigarettes are significantly higher than among adults. However, the proportion of African American adolescent cigarette smokers (71.9%) reporting smoking menthol cigarettes is significantly lower than the corresponding proportion for African American adult smokers (82.7%). This observed difference requires additional study to determine if it is a real difference or an artifact of misreporting.

#### Racial/ethnic group

Big racial/ethnic differences exist in menthol cigarette use. Very high proportions of black/African American adolescent smokers smoke menthol cigarettes [13,25-27]. Recent national data shows that about seven of ten African American smokers in this age group reported smoking menthol cigarettes, followed by about more than half of multi-race and Asian adolescent smokers (Figure 2). Data from the NSDUH survey shows that among adolescent smokers aged 12-17 years, 51.5% of Asians, 47.0% of Hispanics, and 41.4% of Native Hawaiians/Pacific Islanders reported smoking a menthol brand in the past 30 days. Similarly, a study conducted by Appleyard [13] and colleagues using 2000 NYTS data showed that 58.0% of Asians reported using a menthol cigarette brand. However, in another study conducted by Moolchan [19] in Baltimore, MD he found no difference in menthol cigarette use between blacks/African Americans (99.3% and 97.6% for males and females, respectively) and whites (92.3% and 87.5%, respectively). This study, however, used a convenience sample. Appleyard and colleagues also found that high school black/African American, Asian, and Native Hawaiian and Pacific Islander smokers were more likely to report smoking menthol cigarettes than their counterparts in middle school; while white and Hispanic high school smokers were less likely to report smoking menthol cigarettes than their counterparts in middle school [13]. A similar finding for whites and Hispanics (less smoking of menthol cigarettes among high school students) was found by Hersey and colleagues [14].

#### Gender

No consistent gender difference in menthol cigarette use is observed among adolescents. The scientific literature provided found no gender difference in menthol cigarette use between male and female adolescent smokers. Giovino [9], Hersey [14], Kaufman [15], Cummings [18], Johnston [21] found no gender difference between males and females who smoked the menthol brand Newport™*or reported smoking menthol cigarettes in general. The use of trade names is for informational purposes only and in no way implies endorsement by the US Government, the US Department of Health and Human Services, or the US Centers for Disease Control and Prevention. Additional analyses were performed using data from the NSDUH, NYTS, and MTFS. NSDUH data showed that girls aged 12-17 years were more likely to smoke menthol cigarettes than boys the same age in years 2004, 2005, and 2006, but no gender difference was observed in 2007 and 2008 (Table 1). When gender difference for this age group was assessed looking at the youth menthol leading cigarette brand Newport™, no differences were observed in any of these years (data not shown). Using NYTS data for years 2004, 2006, and 2009 combined, no gender difference in menthol cigarette use was observed in middle school (47.9% for females, 50.9% for males), however, a gender difference was observed in high school students, with females more likely to smoke menthol cigarettes than males (p-value= 0.0001) (Table 2). When data for all 3 years were combined, 49.2% of high school girls reported smoking menthol cigarettes compared to 41.0% of high school boys (results not shown). However, no differences were observed in using Newport™ between males and females when using 2004, 2006, and 2009 combined NYTS data (results not shown). MTFS for years 2004-2009 found no gender difference in smoking Newport™ among 8^th^, 10^th^, or 12^th^ graders (data not shown).

#### Brand preferences

**Figure 3:**
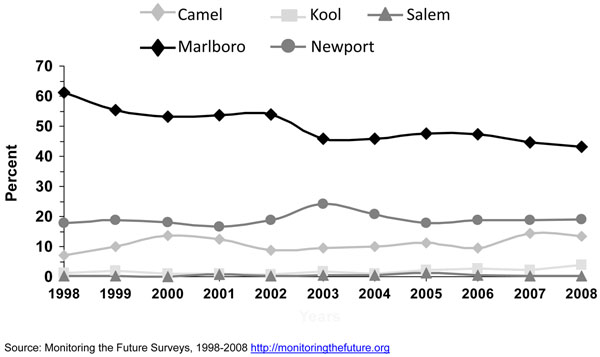


**Figure 4:**
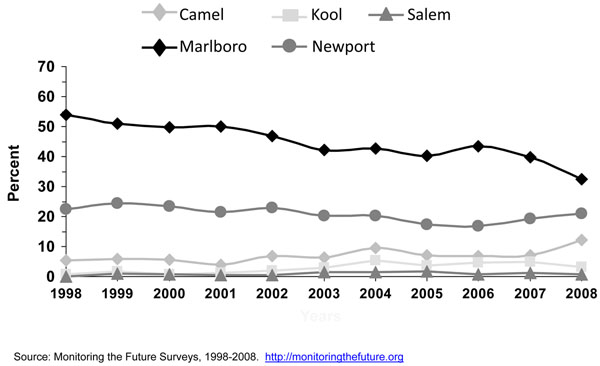


**Figure 5:**
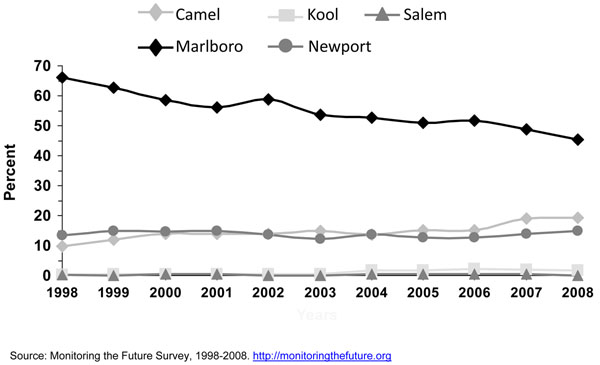


Research has shown that cigarette brand use varies by age, gender, and race/ethnicity [23,24,29,30]. Since efforts were initiated in specific studies in the late 1980’s [15,16] and the early 1990’s [18] to assess cigarette brand preference among adolescent smokers, Marlboro™, Newport™, and Camel™ were the top three cigarette brands smoked by adolescent smokers. Kaufman and colleagues [15] stated that even though market shares for the cigarette brands Marlboro™ and Camel ™changed little between 1989 and 1996, the prevalence of smoking Newport™ cigarettes doubled among white and Hispanic adolescents who usually bought their cigarettes during that period. Barker and colleagues [16] also found that the prevalence of smoking Newport™ cigarettes increased among adolescents from 1989 to 1993, and also for Camel™, while the prevalence of smoking Marlboro™ cigarettes decreased during this period. Consistent with results from earlier years, the 1999 MTFS report [21] showed that Marlboro™ was the predominant brand for 8^th^, 10^th^, and 12^th^ graders, ranging from 53.7%, 61.1%, to 65.2%; followed by the menthol brand Newport™ (22.5%, 17.7%, and 13.3%, respectively). The third-ranked brand was Camel™ (5.4%, 7.3%, and 9.6%, respectively). Also consistent with earlier studies [15,18] big racial/ethnic differences were found. While the great majority of white (61-70%) and Hispanic (57-65%) smokers smoked Marlboro™, the vast majority of blacks/African Americans smoked Newport™ (71-82%). Similar results were observed by Appleyard and colleagues [13] using 2000 NYTS data for middle and high school students. Appleyard found that the vast majority of white, Hispanic, Asian, and Native Hawaiian/Pacific Islander adolescent smokers smoked Marlboro™, and the vast majority of black/African American adolescent smokers smoked Newport™. The predominance of these three cigarette brands still persist [24]. Figures 3 to 5 using 1998 to 2008 MTFS surveys show that the three leading cigarette brands smoked by adolescents are Marlboro™, Newport™, and Camel™, in that order. A 2005 NSDUH report on cigarette brand preference showed that 81.3% of smokers aged 12-17 years and 82.4% of smokers aged 18 to 25 years smoked one of the top 3 brands [24]. Those 26 years or older reported to smoke a somewhat more diversified selection of cigarette brands, only 54.1% of smokers smoked one of the top 3 brands.

#### Geographic Differences in Cigarette Brand Use Preferences

Data on use of menthol cigarettes by region they live in the U.S. is scarce. Compared to the East region of the United States, Kaufman and colleagues [15] found in the 1990’s that adolescents living in the Midwest, South, and West were less likely to smoke Newport™ and more likely to smoke Marlboro™.

#### Smoking initiation

It has been hypothesized that menthol cigarette brands play an important role in smoking initiation. When looking at three age groups (12-17 years, 18-25 years, 26 years or older), the younger the age group, the more likely it is to report smoking menthol cigarettes [10]. Unfortunately, data on this topic is scarce and data from cohort studies are lacking. It is important to clarify that the age of a smoker and smoking initiation is not equivalent. A person that is younger may have been smoking longer than an older person. For example, an adolescent aged 14 years may have been smoking for 3 years, while a young adult aged 19 years may have started smoking less than 1 year ago. Thus, age or age group (12-17 years, 18-25 years, 26 years or older) of the smoker is not equivalent to smoking initiation. The best study design to assess if adolescents and young adults are starting to smoke with a menthol cigarette brand is a prospective/cohort study. However, no cohort study we are aware of collects this type of information for persons younger than age 18 years, when most smoking initiation happens.

Due to lack of information on smoking initiation with menthol cigarettes from prospective studies, and in an attempt to determine if smoking initiation is correlated with menthol cigarette use, we conducted an analysis using cross-sectional data to determine the prevalence of self-reported menthol cigarette use at different “stages” or trajectories of cigarette smoking among adolescents in grades 6^th^ – 12.^th^ This type of analysis has been used to determine the prevalence of nicotine dependence at different stages/trajectories of cigarette smoking among adolescents [31]. Although this approach is inferior to collecting data from prospective studies, it is also true that patterns of cigarette smoking develop over months or years within individuals. In fact, most new smokers take an average of 2-3 years from the time they smoked their first cigarette to the time they become daily smokers [32]. Thus, the analysis presented here for respondents who started smoking less than 2 years ago may give us an insight on the relation of smoking initiation (earliest stages/trajectories of smoking) and the use of menthol cigarettes. We combined data from the 2004, 2006, and 2009 National Youth Tobacco Survey (NYTS). When looking at potential “stages” or trajectories of cigarette smoking and the use of menthol cigarettes, no significant differences were observed between “stages” or trajectories and the prevalence of smoking menthol cigarettes (Table 3). Among adolescents who smoked <1 cigarette per day (CPD) on 1-5 days of the past 30 days, 39.9 percent reported smoking menthol cigarettes. For those who smoked 1-5 CPD on 1-5 days, 6-9 days, 10-19 days, 20-29 days, and all 30 days, the self-reported prevalence of smoking menthol cigarettes among all smokers was 45.3%, 47.5%, 44.2%, 49.7%, and 46.6%, respectively. Among those smoking all 30 days, 42.7% of those who smoked 6-10 CPD, 43.3% of those who reported smoking 11-20 CPD, and 64.9% of those who smoked 20 or more CPD said they smoked a menthol brand. Thus, if smoking a menthol cigarette is a factor associated with smoking initiation, it would be expected to see a higher proportion of menthol cigarette smokers at the earlier stages/trajectories of cigarette smoking. In fact, we found the prevalence of menthol use to be lower among those who smoked <1 CPD on 1-5 days than those who smoked 1-5 CPD on 1-5 days or 1-5 CPD on 20-29 days and to be similar to those in other stages/trajectories of cigarette smoking. Also, those at the highest level of cigarette smoking (20+ CPD on all 30 days) were more likely to report smoking menthol cigarettes than all other smoking stages/trajectories. Thus, we found no indication that adolescent smokers are more likely to initiate smoking by smoking menthol cigarettes.

**Table 3:** Percentage of respondents who reported smoking menthol cigarettes by quantity and frequency of past month cigarette use (2004, 2006, and 2009 combined NYTS).

Days smoked P30D	Cig day	Menthol	Row Percent	Lower 95% Limit ROWPER	Upper 95% Limit ROWPER	Sample Size	Weighted Size
1-5 days	< 1 CPD	Menthol	39.92	37.41	42.48	794	245,029
		Non-menthol	60.08	57.52	62.59	1109	368,755
	1-5 CPD	Menthol	45.32	42.73	47.94	999	333,274
		Non-menthol	54.68	52.06	57.27	1145	402,075
6-9 days	1-5 CPD	Menthol	47.47	41.73	53.27	334	111,694
		Non-menthol	52.53	46.73	58.27	353	123,604
10-19 days	1-5 CPD	Menthol	44.2	38.81	49.74	419	140,144
		Non-menthol	55.8	50.26	61.19	466	176,911
20-29 days	1-5 CPD	Menthol	49.69	44.12	55.26	387	150,554
		Non-menthol	50.31	44.74	55.88	370	152,443
30 days	1-5 CPD	Menthol	46.57	40.59	52.65	396	139,479
		Non-menthol	53.43	47.35	59.41	375	160,050
	6-10 CPD	Menthol	42.72	36.42	49.27	323	118,387
		Non-menthol	57.28	50.73	63.58	344	158,725
	11-20 CPD	Menthol	43.33	36.06	50.9	196	80,015
		Non-menthol	56.67	49.1	63.94	209	104,659
	20+ CPD	Menthol	64.87	59.38	70	283	94,965
		Non-menthol	35.13	30	40.62	136	51,424

#### Trends in menthol cigarette use

**Figure 6:**
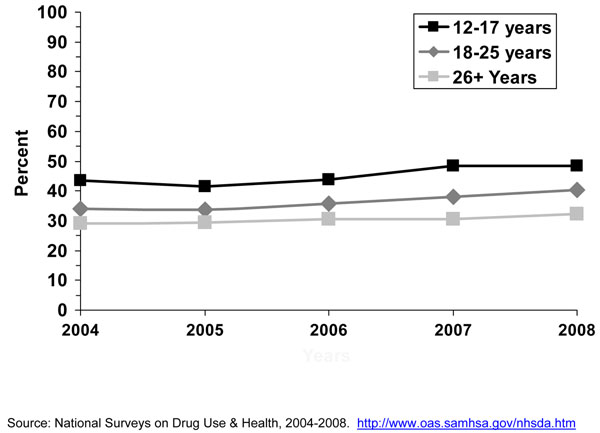


**Figure 7:**
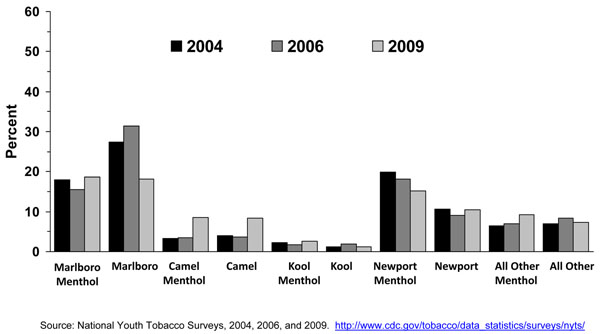


**Figure 8:**
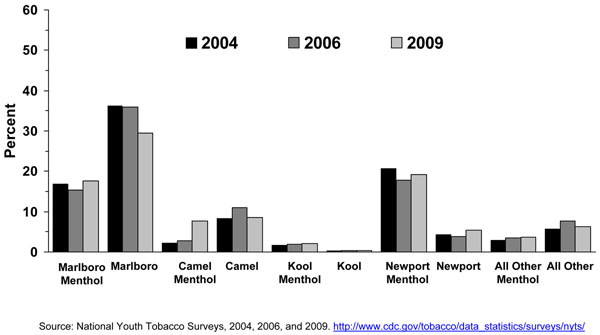


In general, the prevalence of cigarette smoking in the U.S. has been declining for adolescents over the past 10 years. The Youth Risk Behavior Survey (YRBS) shows that cigarette smoking among 9^th^-12^th^ grade students fell by 44% from 1997 to 2007, from 36.4% to 20.0%, a percentage point decline in smoking prevalence of 16% [32], while the proportion of adolescent cigarette smokers reporting to smoke menthol cigarettes has increased significantly from 2004 to 2008 (Figure 6). Among all past-month smokers aged 12-17 years, the proportion of smokers reporting smoking menthol cigarettes increased significantly from 43.4% in 2004 to 48.3% in 2008, for an 11% increase over 4 years. This increase in the proportion of adolescent cigarette smokers who smoked menthol cigarettes reflects an increase in menthol cigarette use among white adolescents (40.3% to 46.0%), who were the only racial/ethnic group to show a significant increase over this period (results not shown). Looking at trends of some specific cigarette brands using MTFS data from 1998 to 2008, no consistent or significant change was observed during this period for Newport™, a predominantly menthol brand, among 8^th,^ 10^th^, and 12^th^ graders, however, a significant increase was observed for Kool™, another menthol brand, for 10^th^ and 12^th^ graders (Figures 3, 4, 5). Similarly, using data from the 2004, 2006, and 2009 NYTS survey, a slight nonsignificant decrease in smoking Newport™ was observed among middle school smokers and no change among high school smokers (Figures 7 and 8). The lack of significant changes for specific cigarette brands over time may be due, at least in part, to less precision of the estimates due to smaller sample size numbers.

Camel™ and Marlboro™ cigarette brands come both in menthol and nonmenthol. The MTFS survey does not distinguish however between these two types of cigarettes. The MTFS data from 1998 to 2008 showed a significant increase in Camel™ use for boys and girls in 10^th^ and 12^th^ grade and declines in Marlboro™ use among smokers in 8^th^, 10^th^, and 12^th^ grade (Figures 3 to 5). A decline in Marlboro™ use was also observed among middle and high school boys and girls in the 2009 NYTS compared to previous years (data not shown). However, because most of the cigarette market share is driven by adult smokers, this decline may not be reflected in the overall Marlboro™ market share. Finally, Kreslake and colleagues reported that expenditures in advertising for nonmenthol brands declined from $309.3 million in 1998 to $39.8 million in 2005 while expenditures in advertising for menthol brands increased from $36.5 million to $43.8 million during that period [8]. It is known that adolescents are highly susceptible to advertising influences. Tobacco advertising, marketing, and promotion play an important role in increasing smoking initiation and tobacco use among youth [34]. Youth have been purposely targeted through advertising, marketing, and promotion [34]. The total weight of evidence presented by the 2008 NCI’s Monograph #19, which was assessed from multiple study types including experimental, cross-sectional, and longitudinal studies, and conducted by investigators from different disciplines using data from several countries, demonstrated a causal relationship between tobacco advertising, marketing, and promotion that ultimately increased smoking initiation and tobacco use [34]. It is possible that increases in expenditures in advertising for menthol brands resulted in increases in youth exposure to such advertising, which then resulted in increase prevalence of use of menthol cigarette brands.

### Adults

About three out of ten adult cigarette smokers reported smoking a menthol cigarette brand (Figure 1). Young adult smokers aged 18-25 years are more likely to smoke menthol cigarettes than older adult smokers (aged 26 years or older). Figure 1 shows that a higher proportion of cigarette smokers aged 18-25 years smoked menthol cigarettes (36.5%) than older adults (30.1%).

#### Racial/ethnic group

Among adult smokers, the vast majority of black/African American adults reported smoking a menthol cigarette brand [18,22,35-39]. Other ethnic groups that may have a high proportion of menthol cigarette smokers are Puerto Ricans [37], Asians [39], and Native Hawaiians/Other Pacific Islanders (Figure 2). More recent information using data from the NSDUH survey shows that about eight of ten black/African American adult smokers reported smoking menthol cigarettes, followed by about half of Native Hawaiian and Other Pacific Island adult smokers (Figure 2). In fact, almost half of adult menthol cigarette smokers are from minority racial/ethnic groups. Analyses with this NHANES data confirm the NSDUH result that most black/African American smokers smoke a menthol brand (results not shown). Thus, one consistent finding is that black/African American smokers are far more likely to smoke a menthol cigarette brand than smokers of other U.S. racial/ethnic groups, however, some other racial/ethnic groups of smokers such as Hawaiians and Other Pacific Islanders and probably Puerto Ricans also smoke menthol cigarettes in high proportion [37,39].

#### Gender

**Figure 9:**
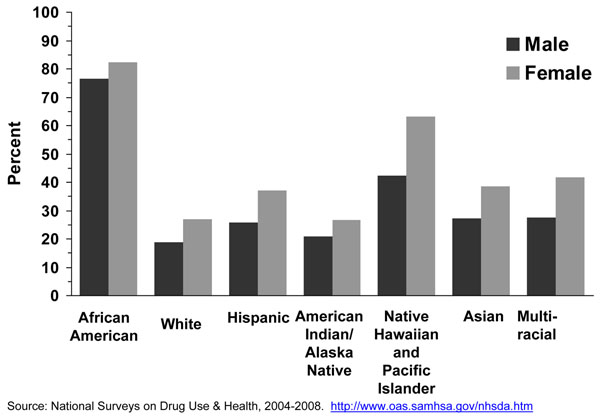


Several studies [18,22,35-39] and further data analyses performed using recent NSDUH (2004-2008) and NHANES (2003-2008) data confirm that female adult smokers are more likely to smoke menthol cigarettes than male adult smokers (Table 4). The scientific literature is consistent in this finding for adult smokers but not for adolescent smokers. NSDUH (2004-2008) data shows that a higher proportion of adult female smokers than adult male smokers reported smoking menthol cigarettes. This was observed for both 18-25 years and 26 years or older. The gender difference was also observed among African Americans, whites, and Hispanics (Figure 9). The lack of a significant gender difference for the other racial/ethnic groups probably resulted from the lack of precision of the estimates for these populations due to small sample size. However, no gender difference was observed for the menthol brand Newport™ when using 2004-2008 NSDUH data (data not shown). Further analysis using NHANES 2003-2008 data in which smokers showed their pack of cigarettes to the interviewer also showed a gender difference where female adult smokers aged 26 years or older were more likely to smoke a menthol brand than their male counterpart, but not so among the age group 20-25 years old, probably due to smaller sample size number of smokers in the survey in this age group (results not shown). No gender difference in Newport™ use was observed between males and females in the age groups 20-25 years or 26 years or older in NHANES (results not shown).

**Table 4:** Proportion of current smokers who use menthol cigarettes by gender and age, NHANES 2003-2008, combined

AGEGRP	Gender	Menthol	Row Percent	Lower 95% Limit ROWPER	Upper 95% Limit ROWPER	Sample Size	Weighted Size
20-25	Male	Menthol	28.07	19.84	38.10	56	784,882
		Non-Menthol	71.93	61.90	80.16	100	2,011,301
	Female	Menthol	34.4*	24.12	46.38	47	644,391
		Non-Menthol	65.6	53.62	75.88	70	1,228,981
26+	Male	Menthol	20.05	16.95	23.56	303	3,212,510
		Non-Menthol	79.95	76.44	83.05	854	12,806,029
	Female	Menthol	35.06	30.97	39.38	363	5,166,768
		Non-Menthol	64.94	60.62	69.03	568	9,569,979

* Estimate may be unreliable due to small sample size (less than 50 participants in cell).

#### Brand preferences

**Figure 10:**
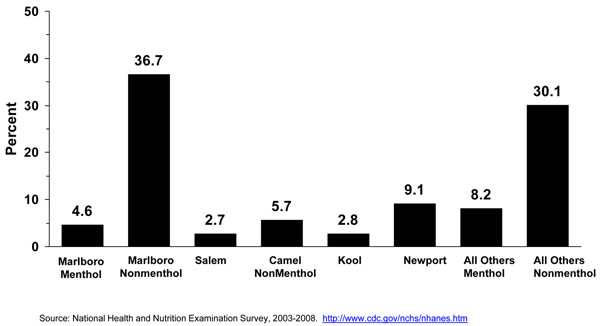


We mentioned earlier that cigarette brand use varies by age and that cigarette brands selection is more diversified for adults aged 26 years or older than those aged 12-17 years and 18-25 years old. The 2005 NSDUH report showed that 54.1% of smokers’ aged 26 years or older smoked one of the top 3 brands (Marlboro™, Newport™, Camel™) compared to 81.3% of smokers aged 12-17 years and 82.4% of smokers aged 18 to 25 years (24). Table 5 and Figure 10 show the distribution of specific menthol and nonmenthol brand use by smokers aged 20 years or older verified by the UPC code. The percent of menthol smokers among all smokers for 2003-2008 was 27.4%. This is similar to reported market share data for years 2003-2005 [1], where menthol brands represented about 26% to 27% of the market share of cigarettes; except for 2006, when it was reported to be 20%. Both Table 5 and Figure show that among specific menthol brands, Newport™ is the leading menthol brand smoked by adult smokers (7.8% - 11.6%), followed by Marlboro Menthol™ (3.9% - 5.9%), Kool ™ (2.4% - 3.2%), and Salem™ (1.2% - 3.2%). When menthol brands other than the ones mentioned above were combined, they represented about 7.0% - 8.7% of the brands smoked by all adult smokers. Marlboro™ nonmenthol brands (33.3% - 39.8%) and nonmenthol brands (27.2% - 33.0%) other than Camel™ represented the vast majority of brands smoked by adult smokers.

**Table 5:** Menthol brand use assessed with UPC bar code among adult smokers aged 20 years or older, NHANES 2003-2008.

5 Common Brand + Menthol info	Column Percent	Lower 95% Limit ROWPER	Upper 95% Limit ROWPER	Sample Size	Weighted Size
Marlboro Menthol	4.60	3.92	5.94	85	1621802
Marlboro (non-Menthol)	36.67	33.33	39.82	763	12924262
Salem (Menthol)	2.73	1.21	3.23	60	960995
Camel (non-Menthol)	5.75	5.24	6.51	115	2026065
Kool (Menthol)	2.81	2.43	3.26	90	992030
Newport (Menthol)	9.11	7.83	11.61	319	3211101
Menthol- All Other Brands	8.19	7.02	8.71	208	2887529
Non-Menthol- All Other brands	30.13	27.27	33.03	710	10620073

**Table 6:** Prevalence for menthol by gender and age, NSDUH 2004-2008, by Year

YR	Age	GENDER	Menthol	Row Percent	Lower 95% Limit ROWPER	Upper 95% Limit ROWPER	Sample Size	Weighted Size
2004	18-25	Male	Menthol Cigarettes	31.61	29.24	34.07	1184	2237546
			Non-menthol cigs	68.39	65.93	70.76	2568	4841562
		Female	Menthol Cigarettes	37.14	35.48	38.83	1336	2102097
			Non-menthol cigs	62.86	61.17	64.52	2298	3557619
	26+	Male	Menthol Cigarettes	24.91	23.04	26.88	676	5918660
			Non-menthol cigs	75.09	73.12	76.96	2080	17838916
		Female	Menthol Cigarettes	34.11	31.57	36.74	883	6900200
			Non-menthol cigs	65.89	63.26	68.43	1700	13331830
2005	18-25	Male	Menthol Cigarettes	31.45	29.66	33.3	1211	2198245
			Non-menthol cigs	68.55	66.7	70.34	2492	4790742
		Female	Menthol Cigarettes	36.46	34.07	38.92	1344	2051208
			Non-menthol cigs	63.54	61.08	65.93	2147	3574756
	26+	Male	Menthol Cigarettes	24.04	21.69	26.55	658	5854296
			Non-menthol cigs	75.96	73.45	78.31	2029	18501160
		Female	Menthol Cigarettes	35.41	33.25	37.64	966	7461473
			Non-menthol cigs	64.59	62.36	66.75	1743	13607506
2006	18-25	Male	Menthol Cigarettes	33.45	31.29	35.69	1276	2300136
			Non-menthol cigs	66.55	64.31	68.71	2440	4575741
		Female	Menthol Cigarettes	38.22	36.02	40.46	1278	2161662
			Non-menthol cigs	61.78	59.54	63.98	2007	3494550
	26+	Male	Menthol Cigarettes	26.72	24.51	29.05	705	6610475
			Non-menthol cigs	73.28	70.95	75.49	1979	18129719
		Female	Menthol Cigarettes	34.86	32.37	37.43	899	7390567
			Non-menthol cigs	65.14	62.57	67.63	1728	13811082
2007	18-25	Male	Menthol Cigarettes	35.9	33.72	38.14	1289	2368757
			Non-menthol cigs	64.1	61.86	66.28	2307	4229075
		Female	Menthol Cigarettes	41.01	38.8	43.27	1349	2095324
			Non-menthol cigs	58.99	56.73	61.2	1857	3013481
	26+	Male	Menthol Cigarettes	27.18	24.7	29.8	746	6668036
			Non-menthol cigs	72.82	70.2	75.3	2010	17868170
		Female	Menthol Cigarettes	34.69	32.59	36.86	940	7217388
			Non-menthol cigs	65.31	63.14	67.41	1733	13586371
2008	18-25	Male	Menthol Cigarettes	38.1	35.79	40.47	1456	2457119
			Non-menthol cigs	61.9	59.53	64.21	2275	3991319
		Female	Menthol Cigarettes	43.12	40.7	45.57	1414	2186195
			Non-menthol cigs	56.88	54.43	59.3	1816	2884040
	26+	Male	Menthol Cigarettes	28.05	25.45	30.82	736	6773295
			Non-menthol cigs	71.95	69.18	74.55	1825	17370270
		Female	Menthol Cigarettes	36.87	34.04	39.79	965	7813637
			Non-menthol cigs	63.13	60.21	65.96	1665	13381077

#### Geographic differences in cigarette brand use

Data on use of menthol cigarettes by region they live in the U.S. is also scarce for adults. A table using 2000 NSDUH data showed that smokers living in the Northeast of the United States were more likely to report smoking menthol cigarettes [9] than those living in the South, North Central, and West of the United States. Similarly, a 2005 NSDUH report on brand preferences confirmed that 18.2% of smokers aged 12 years or older, the vast majority of them adult smokers, and living in the Northeast reported smoking Newport™ compared to 10.7%, 12.4%, and 3.3% in the Midwest, South, and West of the United States, respectively [24].

#### Income

**Figure 11:**
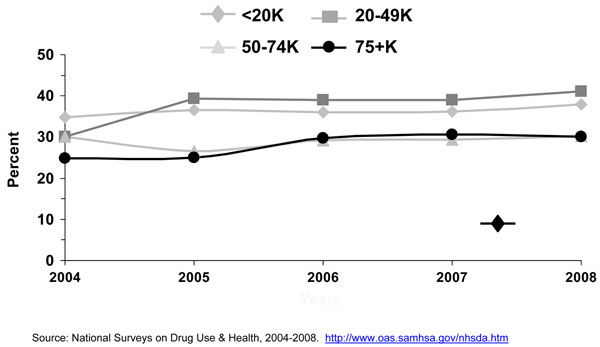


Data on income and the use of menthol cigarettes is almost nonexistent in the scientific literature. A data analysis using 2004-2008 NSDUH showed that adult smokers with family incomes of less than $50,000 were more likely to smoke menthol cigarettes than adult smokers with higher family incomes (Figure 11).

#### Trends in menthol cigarette use

**Figure 12:**
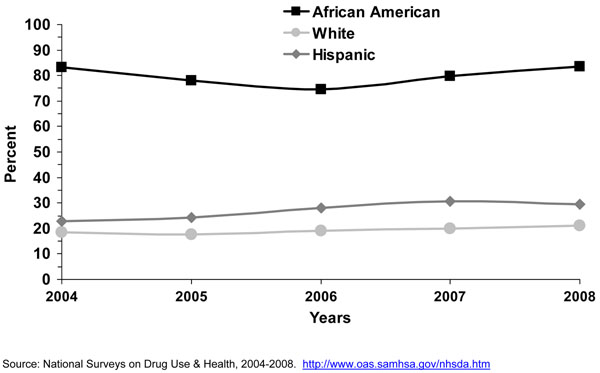


**Figure 13:**
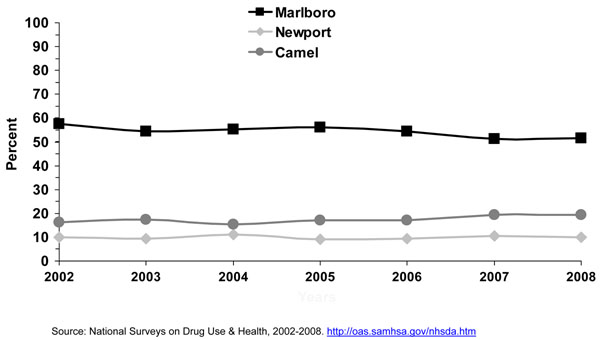


**Figure 14:**
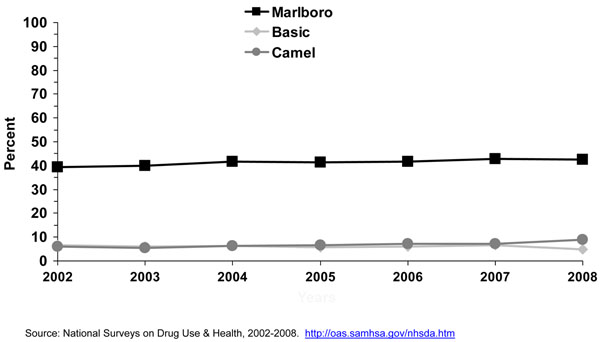


From 2004 to 2008, the proportion of menthol cigarette smokers among all cigarette smokers increased among adults aged 18-25 years and 26 years or older (Figure 6). The proportion of cigarette smokers who reported smoking menthol cigarettes increased significantly from 2004 to 2008 among white and Hispanic men (Figure 12). No changes were observed for white, black/African American, and Hispanic women (results not shown). The lack of significant changes for black African American men and women may be explained, at least in part, to a ceiling effect, where about 80% of men and 90% of women who smoked already smoked a menthol brand. Looking at trends in cigarette brand use from 2002 to 2008 using NSDUH data, no significant changes of Marlboro™, Newport™, or Camel™ use were observed for young adults aged 18-25 years (Figure 13), nor for smokers aged 26 years or older (Figure 14). The lack of significant changes for specific cigarette brands over time may be due, at least in part, to less precision of the estimates due to smaller sample size numbers. Kreslake and colleagues reported an increase for Marlboro Menthol™ sales data [8]. They found that although cigarette sales in the U.S. declined 22% from 2000 to 2005, that the sales of menthol cigarettes remained stable and that Marlboro Menthol™ had a consistent market share growth that started in the early 1990’s up to 2006, most recent data available when they published their article. In 2006, the authors reported, Newport™ was the leading menthol brand, followed by Marlboro Menthol™, which they stated was particularly popular among young adult smokers. Using 2004-2008 NSDUH data, we found that for adult smokers, even though the prevalence of cigarette smoking remained the same in 2008 (20.6%) compared to 2004 (20.9%), the self-reported use of menthol cigarettes increased during those years for adults, more so for those aged 18-25 years than those aged 26 years or older.

#### Menthol cigarette use self-report bias

As previously stated, self-reports of types of cigarettes smoked are subject to bias. For example, Hersey [14], Giovino and colleagues [9] as well as Kreslake and colleagues [8] found that menthol cigarette use self-reports are subject to misclassification, probably more so on certain subgroups such as adolescents. Kreslake and colleagues found that 83% of adolescents aged 12-17 years who smoked Newport™, a menthol brand, also reported they were smokers of menthol cigarettes when asked in a separate question, thus 17% of them reported to be smokers of nonmenthol cigarettes which is inconsistent with the menthol brand Newport. When a similar assessment was made for participants aged 35 years or older, they found that only 5% of Newport™ smokers reported in a separate question to be smokers of a nonmenthol brand [8].

Compared to most adult smokers, most adolescent smokers can be considered to be novice smokers. The fact that a substantial proportion of current (smoking at least 1 day of past 30 days, even a puff) adolescent smokers (e.g., first triers, experimenters, <weekly smokers) will not be considered to be an established adult smoker (100 cigarettes smoked or more in lifetime) is likely related to the differential misreporting of the use of menthol cigarettes between the two age groups. For example, a paper published recently by Caraballo and colleagues [31] shows that 26.8% of current adolescent smokers aged 12-18 years started smoking <1 year prior to the time of the interview. In contrast, the vast majority of adult smokers are established smokers, smoke every day (80%), buy their own cigarettes, and most of them smoke at least half-a-pack a day. Learning specific brand characteristics (e.g., full flavor, light or ultra-light; Marlboro Mild™, Marlboro Menthol™, Newport; ™ menthol or nonmenthol) develops over time. It is likely that many adolescent smokers are less knowledgeable than adult smokers of the specific characteristics of the cigarettes they smoke, especially those who are early in the stage/trajectory of cigarette smoking or those who do not (usually) buy their cigarettes. Thus, adolescent smokers are probably more prone to misreport the type of cigarettes they smoke, including a menthol brand, than adult smokers do.

As previously mentioned, collecting information on the type of cigarettes smoked using the 8 or 12 digit UPC bar code information on the side of the cigarette pack may be seen as a solution to the problem of obtaining accurate information on whether the brand smoked by an adolescent is menthol or non-menthol. However, most of the national youth surveys (MTFS, NYTS, YRBS) that collect this type of information are conducted in a school-setting, a place where adolescents who smoke may not bring their cigarettes and where most schools will not allow it. In household surveys (e.g., NSDUH), some of the novice or even experienced smokers may not want their parents/caregivers to know they smoke. Also, they may be less likely than an adult smoker to have a pack of cigarettes with them. There is ample evidence that a degree of self-report bias exists in reporting the use of mentholated cigarettes, especially among adolescents. Assuming that this type of bias is fairly constant over time, the increase over time in menthol cigarette use among adolescents and adults is a real one.

## Conclusion

There is evidence that menthol cigarettes are disproportionately smoked by U.S. adolescents, blacks/African Americans, adult females, those living in the Northeast of the United States, and those with lower family incomes than their counterparts. It is likely that other disparities in menthol cigarette use exist that we have not covered or have not been studied. Based on self-reports of menthol cigarette use, the use of menthol cigarettes among smokers have increased in recent years among adolescents aged 12-17 years, young adults aged 18-25 years, adults aged 26 years or older, white and Hispanic men. No upward trend was observed for predominantly menthol brands such as Newport™, Kool,™ or Salem™, although this may be due, at least in part, due to smaller numbers of smokers who smoked these specific brands or sub-brands of cigarettes, which resulted in less precise estimates.

## Authors’ contributions

RC was the lead scientist on the project and responsible for the intellectual conception and design of the study including the data analysis and interpretation of the data. KA contributed to the conception of the study, conducted the data analysis, and help with the interpretation of the data. KA also helped draft and revise the final manuscript. Both authors have approved the final version of the manuscript for publication.

## Competing interests

The authors declare that they have no competing interests.
